# Injury to infrapatellar branch of saphenous nerve in anterior cruciate ligament reconstruction using vertical skin incision for hamstring harvesting: risk factors and the influence of treatment outcome

**DOI:** 10.1186/s13018-017-0596-x

**Published:** 2017-06-27

**Authors:** Satoshi Ochiai, Tetsuo Hagino, Shinya Senga, Takashi Yamashita, Kotaro Oda, Hirotaka Haro

**Affiliations:** 1 The Sports Medicine and Knee Center, National Hospital Organization, Kofu National Hospital, 11-35 Tenjin-cho, Kofu, Yamanashi 400-8533 Japan; 2 Department of Orthopaedic Surgery, Faculty of Medicine, University of Yamanashi, 1110, Shimokato, Chuo, Yamanashi 409-3898 Japan

**Keywords:** Anterior cruciate ligament injury, Reconstruction, Infrapatellar branch of saphenous nerve, Sensory disturbance, SF-36

## Abstract

**Background:**

Injury to the infrapatellar branch of the saphenous nerve (IPBSN) is a high-frequency complication in anterior cruciate ligament (ACL) reconstruction. We analyzed the risk factor of IPBSN injury in ACL reconstruction. Moreover, we investigated the influence on treatment outcome by this complication.

**Methods:**

One hundred twenty-three patients who underwent ACL reconstruction using semitendinosus tendon graft were studied. Gender, age, BMI, and additional use of gracilis tendon were recorded. Treatment outcome was assessed by Lysholm score, visual analog scale (VAS) pain score, anterior knee pain, knee range of motion (ROM), and the patient-based SF-36. Patients who developed sensory disturbance at 24 months after reconstruction were compared with those without sensory disturbance.

**Results:**

Twenty-six of 123 patients (21.1%) developed postoperative sensory disturbance caused by IPBSN injury. Baseline parameters were not significantly different compared to those in the non-sensory disturbance group. In the sensory disturbance group, treatment outcome evaluated at 24 months post-reconstruction showed Lysholm score of 94.1, VAS of 9.8 mm, anterior knee pain in 7.7%, and limitation of knee extension of 5° in 7.7%. SF-36 scores in all subscales were above the mean national standard scores. Treatment outcome parameters were also not significantly different compared to those in the non-sensory disturbance group, and none of the patients had serious impairment of knee function and activities of daily living.

**Conclusion:**

Injury to IPBSN in ACL reconstruction was not related to age, gender, and physique, and injury frequency did not increase. Evaluation of postoperative treatment outcome showed that IPBSN injury was not related to anterior knee pain or knee ROM limitation, and patients’ subjective evaluation confirmed no serious impact on physical and mental health.

## Background

Anterior cruciate ligament (ACL) reconstruction is associated with relative low risk, because the procedure can be conducted as less invasive arthroscopy-assisted surgery and almost all the patients undergoing reconstruction belong to the healthy young age group. However, comparing to the complication rate of all knee arthroscopic surgeries [1–3], the complication rate of ACL reconstruction was not low [4–6]. ACL reconstruction involves bone invasion, harvest of tendon graft, and retention of internal fixation material, and technical problems during these procedures may contribute to the high complication rate.

Injury to the infrapatellar branch of the saphenous nerve (IPBSN) is the most common complication in ACL reconstruction [7], and high incidence of 22–59% has been reported [8, 9]. This injury occurs during placement of the anteromedial portal for arthroscopic viewing, during harvest of the patellar tendon or medial hamstring tendon, and while drilling the tibial bone tunnel [10–12].

While the prevention of IPBSN injury by paying attention to the skin incision has been discussed, it is difficult to completely avoid the injury [10, 13]. Although several studies suggested that injury to the saphenous nerve and its branches causes relatively mild impairment of activities of daily living (ADL) [13–15], no prospective study that investigates IPBSN injury in detail has been reported. In this study, we hypothesized that IPBSN injury during ACL reconstruction causes some degree of decline in patient satisfaction even if the injury does not result in functional impairment. The purpose of this study was to verify in detail the effects of IPBSN injury on postoperative outcome using conventional treatment evaluation methods together with SF-36, a patient-based evaluation method. Furthermore, for the purpose of finding prevention methods, we also investigated the factors related to IPBSN injury in patients who developed sensory disturbance after ACL reconstruction presumably caused by IPBSN damage.

## Methods

We studied prospectively 123 patients who underwent primary ACL reconstruction using the semitendinosus tendon graft between April 2012 and March 2014 at the Sports Medicine and Knee Center, National Hospital Organization, Kofu National Hospital. This study was approved by the institutional review boards of the Hospital Ethics Committees (registration number 27-8). At 24 months after reconstruction, we examined all patients for sensory disturbance caused by IPBSN injury based on the anatomical distribution of IPBSN and Romanes’s innervation map [16] by pain stimulation using a needle and touch. All sensory examinations were performed by the first author (SO), an orthopedic surgeon. Based on the presence and absence of IPBSN injury, patients were divided into a sensory disturbance group and a non-sensory disturbance group. Three patients who had sensory disturbance presumably caused by injuries other than IPBSN were excluded from analysis. In these three patients, the injury was in the main trunk of the saphenous nerve in one patient and in the sciatic nerve in two patients. Patients who had fractures, injury of other ligaments, and radiographic changes of severe osteoarthritis were also excluded from this study.

The risk factors of IPBSN injury were analyzed by comparing gender, age, BMI, and additional use of gracilis tendon between the sensory disturbance and non-sensory disturbance groups. Furthermore, treatment outcomes were evaluated by comparing preoperative and 24-month postoperative Lysholm score (minimum score 0, maximum score 100; scores below 65 are interpreted as poor function) [17], visual analog scale (VAS) for pain [18], rate of anterior knee pain [19, 20], knee range of motion (ROM), and the patient-based scale SF-36 [norm-based scoring (NBS): the absolute scores of 0–100 are recalculated such that the mean national standard score is 50 and the standard deviation is 10] [21, 22]. Treatment outcome was compared between the sensory disturbance and non-sensory disturbance groups to examine whether IPBSN injury affects treatment outcome. The SF-36 is composed of eight subscales: physical functioning (PF), role-physical (RP), bodily pain (BP), and general health (GH) that are related to physical health, as well as vitality (VT), social functioning (SF), role-emotional (RE), and mental health (MH) that are related to mental health.

### Operative technique [23]

Single-bundle trans-tibial reconstruction was used in all the patients. All surgeries were conducted by the first author (SO) as the main operator.

First, a 1.8- to 2.5-cm longitudinal skin incision was placed on the medial side of the tibial tubercle, and the semitendinosus tendon was harvested. To construct the tendon graft, the harvested semitendinosus tendon was folded four to six times to obtain a bundle with a diameter of 7.5 mm or larger. When the thickness of the prepared semitendinosus tendon was less than 7.5 mm, the gracilis tendon was also harvested. Then the harvested tendon was bundled, and artificial tendons (Endobutton Tape, CL Endobutton, Acufex; Smith & Nephew, Mansfield, Massachusetts) were attached to both ends of the bundle to prepare the tendon graft. From the incision used for harvesting the tendon, a ProTrac ACL guide system (Acufex; Smith & Nephew) was used to produce a tibial tunnel with the same diameter as the tendon graft at the site of ACL insertion to the tibia. Next, in the femoral bone approximately 6 mm anterior to the posterior margin of the intercondylar fossa and at 10 o’clock (for the right knee) or 2 o’clock (for the left knee) position, a femoral tunnel with the same diameter as the tendon graft was made by the trans-tibial method. The tendon graft attached to a guide suture was passed through the tibial tunnel into the femoral tunnel. The femoral side was fixed with Endobutton (Acufex; Smith & Nephew) and the tibial side with spike staples by double stapling. The graft was fixed with the knee flexed at 30° while a tension of 45 N was applied to the tendon graft. All the tendon grafts prepared had diameters of 7.5 mm or larger (7.5–8.5 mm) and lengths of 55 mm or longer (55–65 mm) [24, 25].

### Postoperative management

From day 3 after surgery, range of motion training was started using a hinge brace with angle restriction. Extension was restricted to 20° and flexion to 90° up to 4 weeks after surgery. Partial weight bearing was allowed from 2 weeks, and full weight bearing was allowed at 4 weeks. Competitive sports activities were restarted around 9 months after surgery.

### Concomitant intra-articular injuries

In both groups, concomitant intra-articular injuries were observed under arthroscopic examination during reconstruction (Table 1). There were no significant differences in the rate of concomitant meniscal tears necessitating surgical intervention, the sites of these tears, and the surgical modality between the sensory disturbance and non-sensory disturbance groups. The prevalence of serious cartilage injury graded according to the ICRS classification [26] was also not significantly different between the two groups.

**Table 1:** Complications of anterior cruciate ligament-deficient knees

	Meniscus tear no. of cases (%) [treatment: no. of cases]			Cartilage injury^a^ no. of cases (%) [site of injury with grade: no. of cases]
	Medial	Lateral	Bilateral	
Sensory disturbance group (*n* = 26)	4 cases (15.3%) [PE: 2, R: 2]	8 cases (30.8%) [PE: 6, R: 2]	5 cases (19.2%) medial meniscus [PE: 4, R: 1] lateral meniscus [PE: 3, R: 2]	6 cases (23.1%) medial femoral condyle grade 3: 2, grade 4: 1 lateral femoral condyle grade 3: 0, grade 4: 1 medial femoral and tibial condyles grade 3: 1, grade 4: 1
Non-sensory disturbance group (*n* = 94)	18 cases (18.1%) [PE: 11, R: 7]	32 cases (34.0%) [PE: 18, R: 7]	15 cases (16.0%) medial meniscus [PE: 11, R: 4] lateral meniscus [PE: 8, R: 7]	18 cases (19.1%) medial femoral condyle grade 3: 7, grade 4: 2 lateral femoral condyle grade 3: 3, grade 4: 0 both femoral condyles grade 3: 3, grade 4: 0 medial femoral and tibial condyles grade 3: 1, grade 4: 2

*PE* partial excision, *R* repair

^a^Grade 3 or above in the ICRS classification

### Statistical analysis

Statistical analyses were conducted using Mann-Whitney *U* test, Fisher’s exact test, and two-way ANOVA. A *p* value less than 0.05 was considered significant.

## Results

Among 123 patients who underwent ACL reconstruction, 29 patients developed sensory disturbance, 26 of whom were due to IPBSN injury and 3 were due to other nerve injuries. The prevalence of IPBSN injury in all patients who underwent ACL reconstruction was 21.1% (26 of 123 patients). After excluding the three patients with sensory disturbance due to other causes, the present study compared 26 cases of sensory disturbance due to IPBSN injury and 94 cases without sensory disturbance. Comparison of the two groups showed no significant differences in gender ratio, age, BMI, and the tendons harvested (rate of harvesting both semitendinosus tendon and gracilis tendon) (Table 2).

**Table 2:** Comparison of the group with and the group without sensory disturbance

		Sensory disturbance group (*N* = 26)	Non-sensory disturbance group (*N* = 94)	*p* value
Gender	Male	12	49	0.38
	Female	14	45	
Age (years)		24.7 ± 10.1	24.3 ± 11.9	0.87
BMI		24.6 ± 3.1	23.3 ± 3.7	0.40
Tendon graft^a^		3 patients (11.5%)	10 patients (10.6%)	0.57

*BMI* body mass index

^a^Use of the gracilis tendon together with the semitendinosus tendon in the tendon graft

Apart from sensory disturbance, no re-rupture or laxity of the tendon graft and no serious postoperative complications such as deep infections were observed. In addition, none of the patients dropped out of the postoperative follow-up protocol.

Regarding treatment outcome, Lysholm score and VAS pain score were improved significantly at 24 months after reconstruction compared to before surgery in both groups, but no significant differences were observed between the two groups. The two groups also did not differ in the percentage of patients with anterior knee pain, as assessed based on self-reporting by the patient regarding the site and area of pain in the knee using Spicer’s knee diagram [15]. None of the patients in both groups showed severe pain or occurrence of complex regional pain syndrome (CRPS) (Table 3).

**Table 3:** Evaluations of treatment outcome

		Sensory disturbance group	Non-sensory disturbance group	*p* value
Lysholm score	Preoperative	44.3 ± 29.6	54.2 ± 23.4	0.17
	Postoperative	94.1 ± 6.5	91.6 ± 12.1	0.46
	*p* value Pre vs. post	5.2 × 10^−7^*	2.6 × 10^−13^*	
VAS (mm)	Preoperative	32.5 ± 29.1	43.7 ± 27.7	0.26
	Postoperative	9.8 ± 14.1	13.6 ± 19.6	0.57
	*p* value Pre vs. post	0.036*	2.0 × 10^−6^*	
Rate of AKP		2 patients (7.7%)	2 patients (2.1%)	0.20
ROM limitation		2 patients (7.7%)	5 patients (5.3%)	0.64

Rate of AKP: For the evaluation of acute knee pain, Spicer’s knee diagram was used. The patient self-reported the site of pain on the diagram or the doctor marked on the diagram based on the patient’s information [15]

*AKP* anterior knee pain

*p < 0.05

In the assessment of knee ROM, none of the patients showed a difference in ROM between the affected and unaffected knee (side-to-side difference in ROM) before surgery, because reconstruction was scheduled only when the side-to-side difference in ROM was resolved. The two groups did not differ in the percentage of patients with a side-to-side difference in knee ROM of 5° or more at 24 months after surgery (Table 3). The ROM limitation in all cases was confined to extension only, and no limitation of flexion was observed.

**Fig. 1:**
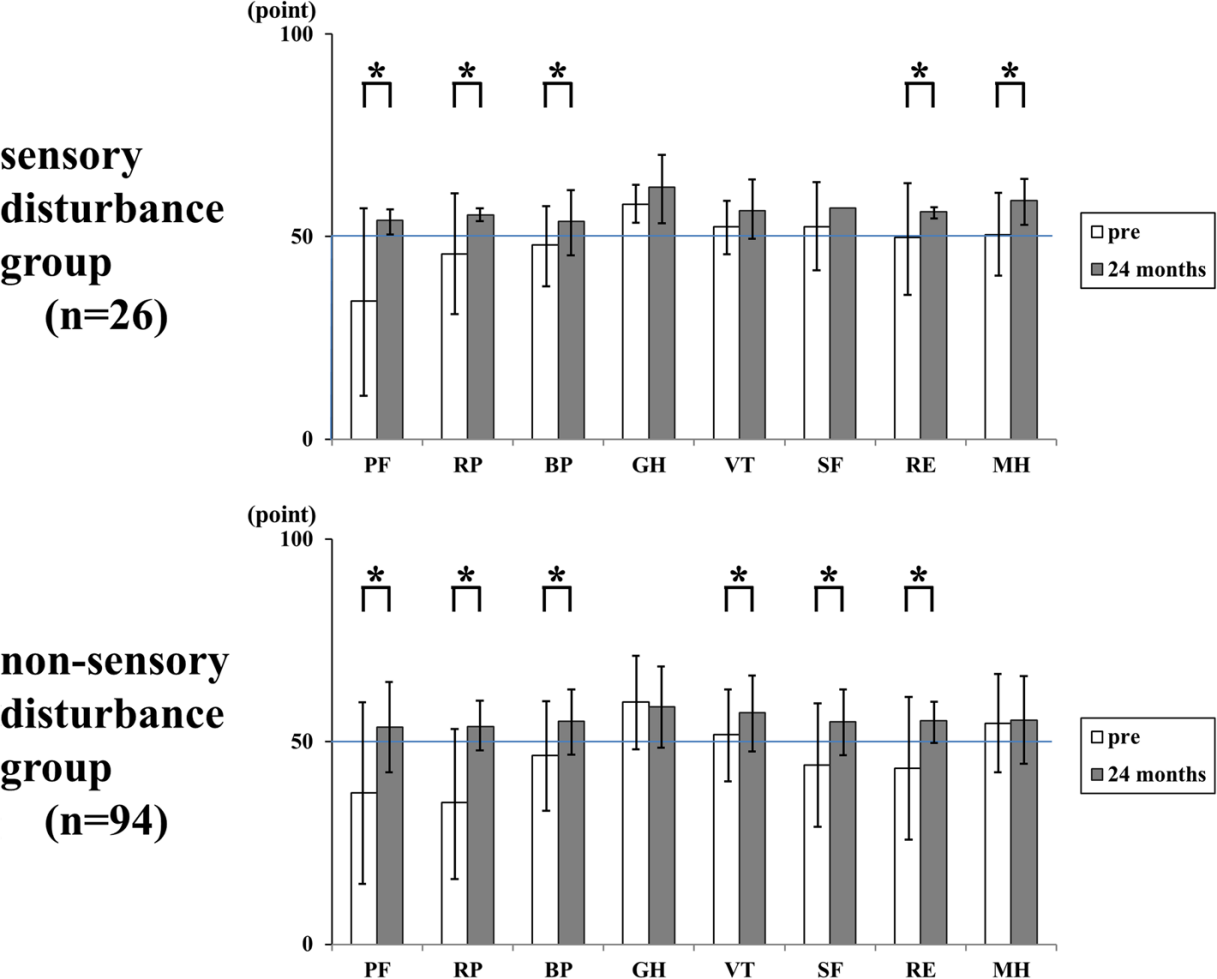
Evaluation using SF-36. *PF* physical functioning, *RP* role-physical, *BP* bodily pain, *GH* general health, *VT* vitality, *SF* social functioning, *RE* role-emotional, *MH* mental health. Preoperative SF-36 evaluation shows scores lower than the Japanese national standard scores in *PF*, *RP*, *BP*, *RE*, and *MH* subscales in the sensory disturbance group, and in *PF*, *RP*, *BP*, *VT*, *SF*, and *RE* subscales in the non-sensory disturbance group. Evaluation at 24 months after surgery shows scores higher than national standard scores in all the subscales in both groups, with no significant differences between the two groups. The asterisk indicates *p* < 0.05. *Horizontal line* at 50 points on the graph = national standard scores in Japan

In preoperative assessment using SF-36, the scores of more than half of the subscales were below the mean Japanese national standard scores in both the sensory disturbance and non-sensory disturbance groups (Fig. 1). At 24 months after reconstruction, the scores of all the subscales were improved to above the mean Japanese national standard scores in both groups, with no significant differences between the two groups.

## Discussion

**Fig. 2:**
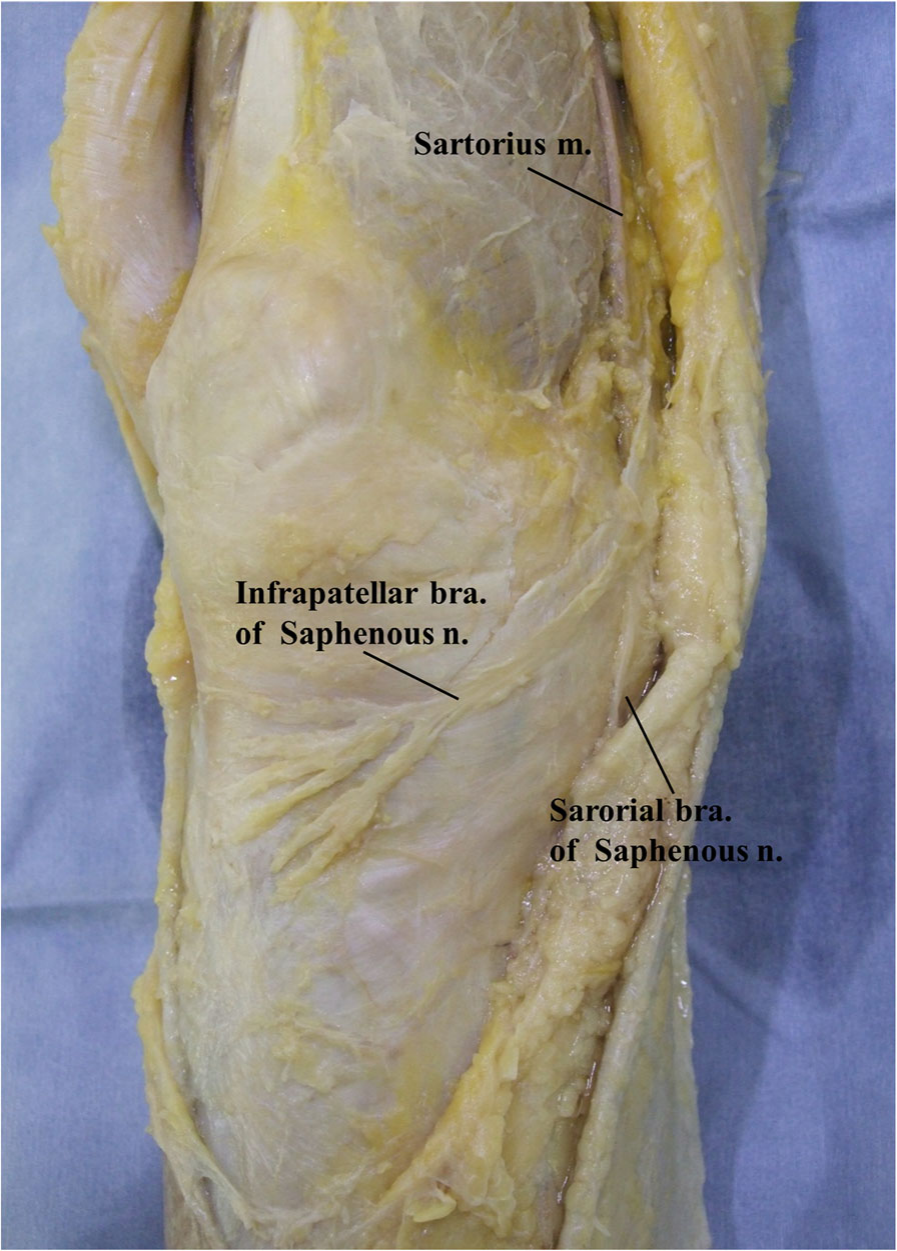
Photograph of dissection of a cadaver showing the course of the infrapatellar branch of the saphenous nerve. The saphenous nerve branches near the medial side of the knee into the infrapatellar branch and sartorial branch. The infrapatellar branch courses at the inferomedial side of the patella. The course has several variations. The positional relationship with the sartorius muscle also varies; the infrapatellar branch may pass over the muscle, under the muscle, or through the muscle. Attention is needed

The saphenous nerve is a purely sensory nerve. The infrapatellar branch of the saphenous nerve traverses the anteromedial aspect of the patella (Fig. 2). Injury to the IPBSN results in the development of sensory disturbance from the anterior knee to the proximal lower leg. This injury requires attention because it occasionally gives rise to anterior knee pain, kneeling pain, painful neuroma, and CRPS [11, 27, 28]. In the present study, we attempted to identify the risk factors associated with IPBSN injury with the aim to help prevent IPBSN injury associated with ACL reconstruction. However, we found no relationship between IPBSN injury and patients’ physical factors of gender, age, and BMI. Regarding surgical factors, Mirzatolooei and Pisoodeh [29] suggested that the risk of IPBSN injury is increased accompanying the harvest of the gracilis tendon, but we found no change in the IPBSN injury rate even when the gracilis tendon was harvested in addition to the semitendinosus tendon. Regarding the skin incision during surgery, the length of skin incision in our study was shorter than the conventional length used in ACL reconstruction, which may account for the lower rate of IPBSN injury (21.1%) compared to other reports (22–59%) [8, 9]. However, Figueroa et al. [30] found no correlation between the presence of sensory disturbance caused by IPBSN injury and the size of the incision or the distance to the tibial tubercle. Crude manipulation of the tendon stripper during harvest of the medial hamstring has been reported to be a cause of IPBSN injury [10]. A similar mechanism may also cause damage to the main trunk of the saphenous nerve and sciatic nerve [11, 31]. There is a possibility that inappropriate manipulation of the tendon stripper may also be involved in IPBSN and other nerve injuries in our institution.

Several techniques have been recommended for the prevention of IPBSN injury, such as performing surgery with the knee in flexion to direct the course of the nerve away from the incision or to use a horizontal or an oblique incision that runs parallel to the course of the nerve [10, 13]. However, even using these methods, the IPBSN injury rates have been reported to range from 14.7 to 43% [9, 32, 33]. Therefore, currently, the IPBSN injury remains a complication that is difficult to avoid in ACL reconstruction.

There are few reports on subjective evaluation of treatment outcome of ACL reconstruction in patients with injury to branches of the saphenous nerve including the infrapatellar branch. Mochizuki et al. [14] investigated skin sensory change after ACL reconstruction using an original questionnaire posted to 60 patients who were followed for a mean of 31.6 months after surgery. They reported that 80.8% of the patients felt no effect on their daily living as a result of the sensory change. Sabat and Kumar [13] evaluated sensory loss prospectively in 120 patients followed for 6 months after surgery using the 2000 IKDC Subjective Knee Evaluation Form. They reported that the sensory loss did not impair normal daily activities. Spicer et al. [15] investigated anterior knee symptoms using the Shelbourne and Trumper [19] anterior knee pain questionnaire distributed clinically or by post or telephone to 44 patients followed for a mean of 30 months after surgery. They found that only 2% of the patients experienced significant symptoms causing limitation of daily activities, although anterior knee symptoms involving the IPBSN occurred commonly. Therefore, the effect of IPBSN injury on treatment outcome should be studied in more detail. In the present prospective 2-year study, we evaluated treatment outcome using the SF-36, which is a patient-based health survey with scientifically proven reliability and validity [34, 35], together with the conventional methods. Our results obtained from the SF-36 survey confirmed good treatment outcome and that patients were not affected by IPBSN injury, from the aspects of both physical and mental health. In addition, evaluation of the Lysholm score, VAS pain score, and knee ROM also revealed favorable treatment outcome with no impact due to IPSN injury. The prevalence of anterior knee pain tended to be slightly higher in patients with IPBSN injury, but the difference was not significant.

The results of the present study demonstrated that IPBSN injury during ACL reconstruction was not related to anterior knee pain or limitation of knee ROM. Subjective evaluation by the patients confirmed that IPBSN injury had no serious impact on physical and mental health. However, since the risk factors related to this injury have not been identified currently, prediction and prevention remain difficult. Considering the possibility of the rare occurrence of CRPS [28, 36], further studies are required.

### Limitations

This study has several limitations. The extent and severity of sensory disturbance were not considered. Since the number of subjects was relatively small, the possibility of occurrence of CRPS and neuroma that have a very low incidence cannot be excluded. Moreover, there is no conclusion regarding measures to prevent IPBSN injury. Further study with a larger study population is required to examine whether changing surgical method ameliorates symptoms of IPBSN injury.

## Conclusion

Injury to IPBSN in ACL reconstruction is not related to age, gender, or physique, and the frequency of injury does not increase even when the gracilis tendon is harvested in addition to the semitendinosus tendon. Regarding treatment outcome after reconstruction, IPBSN injury is not related to anterior knee pain or limitation of knee ROM and has no serious impact on physical and mental health according to patients’ subjective evaluation.

## Abbreviations

ACL: Anterior cruciate ligament
ADL: Activities of daily living
BP: Bodily pain
GH: General health
IPBSN: Infrapatellar branch of the saphenous nerve
MH: Mental health
NBS: Norm-based scoring
PF: Physical functioning
RE: Role-emotional
ROM: Range of motion
RP: Role-physical
SF: Social functioning
SF-36: Patient-based 36-item short-form health survey
VAS: Visual analog scale
VT: Vitality
